# Innovative Pancreas Ligation Band for Distal Pancreatectomy: A Pilot In Vivo Porcine Study

**DOI:** 10.7759/cureus.18238

**Published:** 2021-09-24

**Authors:** Yuji Kaneda, Yuki Kimura, Akira Saito, Hideyuki Ohzawa, Ryusuke Ae, Hiroshi Kawahira, Alan K Lefor, Naohiro Sata

**Affiliations:** 1 Department of Surgery, Jichi Medical University, Shimotsuke, JPN; 2 Medical Simulation Center, Jichi Medical University, Shimotsuke, JPN; 3 Department of Clinical Oncology, Jichi Medical University, Shimotsuke, JPN; 4 Division of Public Health, Jichi Medical University, Shimotsuke, JPN

**Keywords:** postoperative pancreatic fistula, pancreatic necrosis, surgery, porcine study, ligation band, pancreatic stump, distal pancreatectomy

## Abstract

Introduction

Although new techniques and devices have been introduced, the incidence of postoperative pancreatic fistula (POPF) after distal pancreatectomy remains high. To reduce the risk of POPF, we developed an innovative ligation band and conducted this pilot study to assess the possibility of reducing the incidence of POPF and pancreatic necrosis after distal pancreatectomy.

Methods

Distal pancreatectomy was performed in three pigs. In two animals, ligation of the pancreas was performed while maintaining arterial blood flow to the stump, and in one animal, the arterial blood flow was occluded. After ligation, the pancreas was sharply divided. Animals were sacrificed seven days later, and the remnant pancreas was assessed histologically. POPF was defined as amylase in ascites > 3x the preoperative serum amylase level. The following equation was used to quantify the extent of necrotic tissue: necrotic tissue residual rate = necrotic tissue area/ cross-sectional area.

Results

All animals survived, and no POPF developed. For two animals in which arterial blood flow to the stump was maintained, necrotic tissue residual rates at the ligation line were 24% and 31%. At the pancreatic stump, necrotic tissue residual rates were 37% and 50%. In the animal in which arterial blood flow to the stump was occluded, the necrotic tissue residual rate at the ligation line was 83% and that at the pancreatic stump was 78%, both higher than that in animals in which arterial blood flow was maintained. In all animals, there was no injury to pancreatic tissue at the ligation line.

Conclusion

The pancreas ligation band can potentially prevent POPF after distal pancreatectomy by atraumatic ligation, and the band ligates the pancreatic stump while maintaining arterial blood flow and limiting pancreatic necrosis.

## Introduction

Postoperative pancreatic fistula (POPF) is a severe complication in patients after distal pancreatectomy, and the optimal management of the pancreatic transection plane is still debated [1]. POPF can cause serious secondary complications such as false aneurysm, intra-abdominal abscess, and sepsis [2-8], resulting in prolonged hospitalization [2,3,9-11] and increased costs [12,13]. Additionally, POPF is associated with adjuvant chemotherapy failure [14,15], worsening the prognosis in patients with pancreatic cancer [16,17]. Although several new techniques and devices have been proposed to reduce the incidence of POPF after distal pancreatectomy, the incidence remains high at 34-66% compared to other postoperative abdominal complications [4,7,18-20].

We hypothesized that conventional procedures for pancreatic stump closure (e.g., hand-sewn or linear stapler closure) lead to direct injury to pancreatic parenchyma and the pancreatic duct, resulting in an increased risk of developing a POPF. A previous study speculated that tight ligation in hand-sewn closure leads to pancreatic necrosis, leading to the development of POPF [21]. To minimize the risk of POPF, we developed an innovative pancreas ligation band that closes the pancreatic stump without traumatic tissue injury and with adjustable ligation force to maintain arterial blood flow to the pancreatic stump. We conducted a pilot study to assess the possibility of reducing the incidence of POPF and pancreatic necrosis after distal pancreatectomy using the pancreas ligation band.

## Materials and methods

Pancreas ligation band

**Figure 1:**
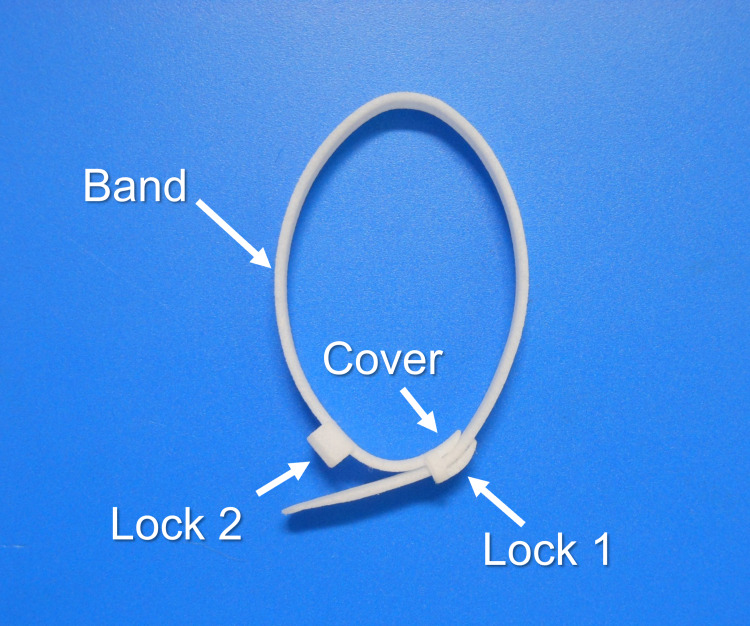
Pancreas ligation band. The ligation band has an adjustable locking system and a cover.

We developed an innovative pancreas ligation band for distal pancreatectomy with technical support from Teijin Medical Technologies Co., Ltd (Osaka, Japan), made from a bioabsorbable polymer that has four specific characteristics (Figure 1). First, the device ligates the distal pancreas without directly traumatic elements, such as needles or staples. Second, an adjustable locking system controls the ligation force applied. Third, a cover on the locking system protects the pancreatic tissue and capsule from becoming caught in the mechanism. Fourth, small projections inside of the device prevent slippage from the pancreatic stump. This pilot study was conducted using a prototype of the device created from plastic with a three-dimensional printer.

Animals

The experimental protocol was approved by the Animal Experiment Committee of our institution (approval no. 18035-02), and all animals were managed according to the ethical regulations for animal studies. Three pigs (two female Landrace pigs and one castrated Yorkshire pig, median [range] weight 26.4 [23.7-35.0] kg) were purchased from Sanesu Breeding Co., Ltd. (Funabashi, Japan).

Distal pancreatectomy

**Figure 2:**
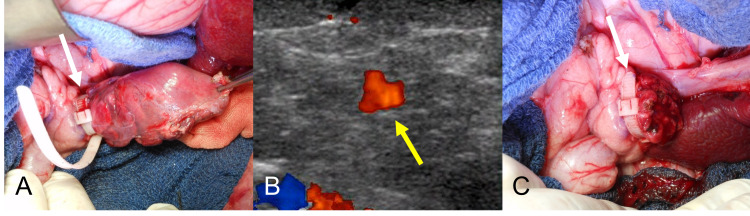
Intraoperative findings. (A) The pancreas is ligated with the pancreas ligation band. (B) Arterial blood flow to the splenic lobe is confirmed by intraoperative ultrasonography. (C) The pancreas is divided at 1 cm to the left of the ligation line. The white arrow indicates the pancreas ligation band. The yellow arrow indicates the arterial blood flow to the splenic lobe.

After overnight fasting, animals were premedicated with medetomidine 0.06 mg/kg, midazolam 0.3 mg/kg, and atropine 0.02 mg/kg intramuscularly. Anesthesia was maintained by endotracheal intubation with sevoflurane, and distal pancreatectomy was performed (Figure 2). The splenic lobe of the pancreas was mobilized through a midline incision, corresponding to the tail and body of the pancreas in the human. Ligation and division locations were set at 1 cm and 2 cm to the left of the confluence of the splenic vein, respectively. The pancreas was ligated while assessing arterial blood flow to the splenic lobe by real-time ultrasonography (Figures 2A, 2B). The systolic blood pressures of animals 1, 2, and 3 while ligation were 109, 90, and 75 mmHg, respectively. In animals 1 and 2, the pancreas was ligated with maximum force within maintaining the arterial blood flow to the stump. In animal 3, the pancreas was ligated with the minimum force needed to occlude arterial blood flow. After ligation, the pancreas was sharply divided and bleeding controlled (Figure 2C). The abdomen was then closed in the routine manner. After surgery, fentanyl transdermal patches (8.4 mg) were placed for 72 hours. Amoxicillin (250 mg/day) was given orally for three days or oxytetracycline (20 mg/kg) by intramuscular administration once. Animals were maintained in individual cages with free access to water and fed once daily. All animals were followed for seven days after surgery, with 100% survival postoperatively.

Assessment of POPF and pancreatic necrosis

Seven days after surgery, animals were anesthetized and the presence of fluid around the pancreatic stump was assessed by computed tomography (CT) scan. The abdomen was then opened and ascites collected. Referring to the International Study Group on Pancreatic Surgery definition [22], POPF is defined as an amylase level in ascites > 3x the preoperative serum amylase level. The remnant pancreas with the band was exposed and harvested. The animals were then euthanized with bolus administration of potassium chloride (60 mg/kg). Specimens were fixed in 10% formalin and processed for routine histologic evaluation. The cross-sectional area and necrotic tissue area in photomicrographs were calculated by ImageJ Fiji (National Institutes of Health, Bethesda, MD). The following equation was used to quantify the extent of necrotic tissue: necrotic tissue residual rate = necrotic tissue area / cross-sectional area.

## Results

CT scan findings

**Figure 3:**
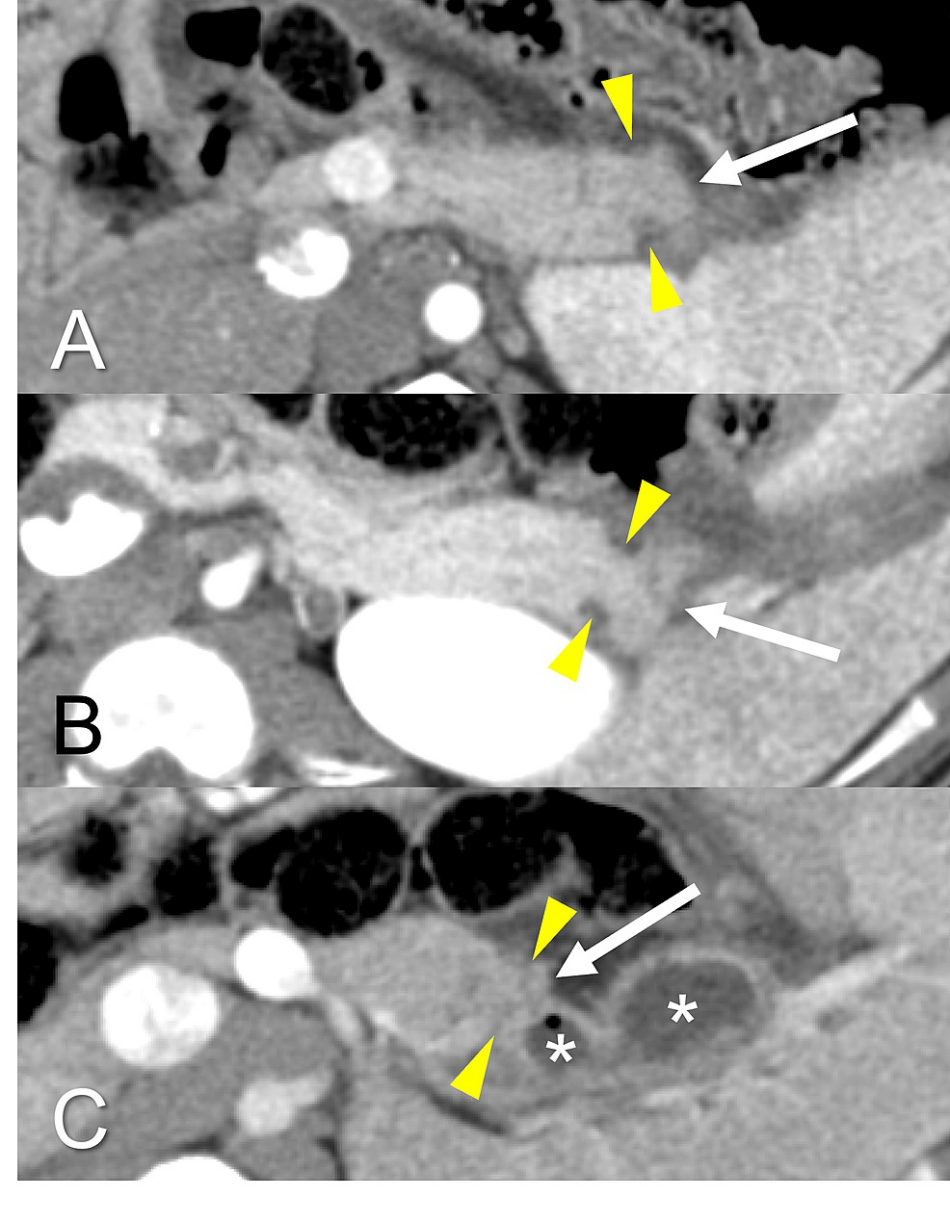
CT scan seven days after distal pancreatectomy. (A) In animal 1, there is no fluid collection around the pancreatic stump. (B) In animal 2, there is no fluid collection around the pancreas stump, similar to animal 1. (C) In animal 3, there is a fluid collection with air around the pancreatic stump. The white arrow indicates the pancreatic stump. The asterisk indicates the fluid collection. The yellow arrowhead indicates the ligation site.

Enhanced abdominal CT scan at seven days after resection and placement of the band are showed in Figure 3. In animals 1 and 2, there was no fluid collection around the pancreatic stumps (Figures 3A, 3B). However, there was a fluid collection with air around the stump in animal 3 (Figure 3C).

Laparotomy findings and assessment of POPF

In all animals, there were a small amount of ascites in the abdominal cavity, and no pancreatic leakage was seen macroscopically. The amylase levels in the ascites in animals 1, 2, and 3 were 816, 1,675, and 810 U/L, respectively. The preoperative serum amylase levels in these animals were 1,156, 2,890, and 1190 U/L, respectively. Given that the amylase levels did not meet the threshold of three times the serum amylase levels, no POPF was diagnosed in all animals. In animals 1 and 2, there were no fluid collections around the pancreatic stump (consistent with the CT scan findings), but a small localized abscess was present in continuity with the pancreatic stump in animal 3, similar to the CT scan findings. No band slippage was observed from the pancreatic stump.

Histological assessment of pancreatic remnant

**Figure 4:**
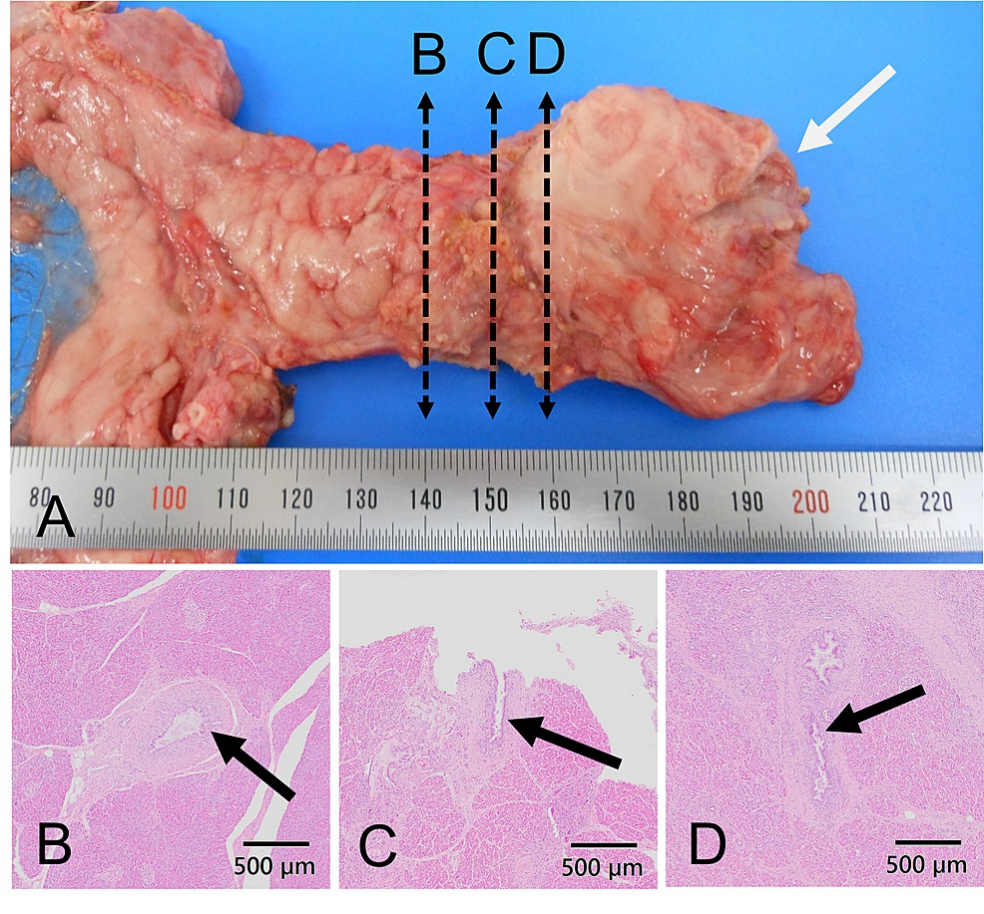
Pathological findings of the pancreas in animal 3. (A) An abscess cavity is grossly present in continuity with the pancreatic stump. (B) In the normal tissue of the pancreas, the main pancreatic duct is patent. (C) At the ligation line, the main pancreatic duct is stenosed. (D) At the stump, the main pancreatic duct is stenosed, similar to the ligation line. The white arrow indicates an abscess cavity. The black dashed arrow indicates the specimen cutting line. The black arrow indicates the main pancreatic duct. Hematoxylin and eosin stain (B-D): 40×.

Gross morphology and pathological findings of the main pancreatic duct are shown in Figure 4. In animal 3, an abscess cavity was present at the pancreatic stump (Figure 4A). The main pancreatic duct was patent in normal tissue but stenosed at the ligation line and the stump in animal 3 (Figures 4B-4D). The findings were similar to those in animals 1 and 2.

**Figure 5:**
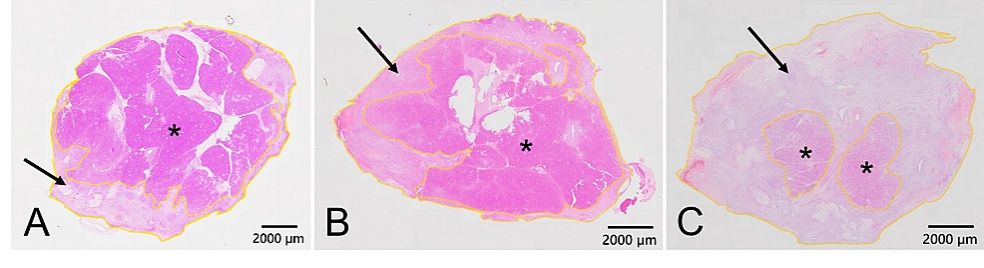
Pathological findings at the ligation line. (A) In animal 1, most pancreatic tissue is preserved, and the extent of necrosis is localized under the capsule. (B) In animal 2, most pancreatic tissue is preserved, similar to animal 1. (C) In animal 3, only a small area of pancreatic tissue is preserved. In all animals, the capsule of the pancreas is preserved, and no injury is detected in subcapsular tissue. The black arrow indicates the necrotic tissue. The asterisk indicates pancreatic tissue. The outer yellow line indicates the outline of the whole tissue. The inner yellow line indicates the border between pancreatic tissue and necrotic tissue. Hematoxylin and eosin stain: 10×.

The pathological findings at the ligation line are shown in Figure 5. In all animals, the capsule of the pancreas was intact without evidence of subcapsular injury. In animal 1, necrotic tissue area and cross-sectional area were 31 mm^2^ and 127 mm^2^, respectively, and necrotic tissue residual rate was 24% (Figure 5A). In animal 2, necrotic tissue area and cross-sectional area were 27 mm^2^ and 86 mm^2^, respectively, and necrotic tissue residual rate was 31% (Figure 5B). Most pancreatic tissue was preserved, and the extent of necrosis was localized under the capsule in animals 1 and 2. In animal 3, without arterial blood flow to the pancreatic stump, necrotic tissue area and cross-sectional area were 79 mm^2^ and 95 mm^2^, respectively, and necrotic tissue residual rate was 83%. Only a small area of pancreatic tissue was preserved in animal 3 (Figure 5C).

**Figure 6:**
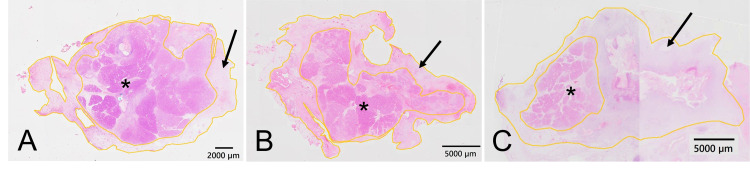
Pathological findings at the pancreatic stump. (A) In animal 1, more than 60% area of the pancreatic tissue is preserved. (B) In animal 2, half the area of the pancreatic tissue is preserved. (C) In animal 3, only around 20% area of pancreatic tissue is preserved. The black arrow indicates the necrotic tissue. The asterisk indicates pancreatic tissue. The outer yellow line indicates the outline of the whole tissue. The inner yellow line indicates the border between pancreatic tissue and necrotic tissue. Hematoxylin and eosin stain: 10×.

The pathological findings at the pancreatic stump are shown in Figure 6. In animal 1, necrotic tissue area and cross-sectional area were 92 mm^2^ and 252 mm^2^, respectively, and necrotic tissue residual rate was 37% (Figure 6A). In animal 2, necrotic tissue area and cross-sectional area were 148 mm^2^ and 296 mm^2^, respectively, and necrotic tissue residual rate was 50% (Figure 6B). More than half of the pancreatic tissue was preserved in animals 1 and 2. In animal 3, necrotic tissue area and cross-sectional area were 268 mm^2^ and 344 mm^2^, respectively, and the necrotic tissue residual rate was 78%. Only a small area of pancreatic tissue was preserved in animal 3 (Figure 6C). The necrotic tissue residual rate in animal 3 was higher than that in animals 1 and 2 at the ligation line and the stump.

## Discussion

This in vivo study has two important findings regarding the use of ligation band for distal pancreatectomy. First, the pancreas ligation band may prevent POPF by atraumatic ligation and the resulting stenosis of the pancreatic duct. Second, the band can ligate the pancreas while maintaining arterial blood flow and reducing pancreatic necrosis.

The pancreas ligation band may prevent POPF by atraumatic ligation and the resulting stenosis of the pancreatic duct. A linear stapler is typically used for distal pancreatectomy to close the pancreatic stump. No significant difference was shown in the occurrence of POPF after distal pancreatectomy comparing linear stapler and hand-sewn closures of the pancreatic stump in the DISPACT trial, a multicenter randomized controlled trial [4]. Several studies have reported the superiority of reinforced staplers over regular stapler closure [23,24], but the occurrence of POPF was still high at 5-16% [19,23-25]. Hand-sewn closure of the pancreatic stump and the stapler both involve penetration of the pancreatic parenchyma and pancreatic duct by needles or staples. We hypothesized that these methods may result in direct injury to pancreatic parenchyma and/or pancreatic ducts, which can cause POPF. The ideal device for distal pancreatectomy would ligate the pancreatic parenchyma and the pancreatic duct atraumatically without needles or staples. The ligation band fulfills this requirement and may be an ideal device for ligation of the pancreatic stump.

The band described here ligates the pancreas while maintaining arterial blood flow and reducing pancreatic necrosis. To date, only one animal study has examined the relationship between ligation and pancreatic necrosis. In this previous study, it was described that ligation using a hand-sewn closure caused necrosis and regenerated pancreatic ducts in mongrel dogs, which may lead to POPF [21]. However, this previous study did not evaluate arterial blood flow to the pancreatic stump during ligation; therefore, the relationship between arterial blood flow and necrosis is unknown. The present study assessed the degree of pancreatic necrosis with assessment of arterial blood flow to the pancreatic stump and shows that maintaining arterial blood flow may reduce pancreatic necrosis. Therefore, we speculate that the ligation band can contribute to minimizing the occurrence of POPF by maintaining arterial blood flow to the stump.

The present study has acknowledged limitations. First, the number of animals evaluated is limited. To accurately evaluate the prevention of POPF and necrosis using the pancreas ligation band, and retention of the band at the pancreatic stump, further experiments are needed. Second, the findings and results obtained after distal pancreatectomy in a porcine model may not be directly generalizable to humans. However, it seems appropriate to use the porcine model because porcine pancreatic parenchyma is similar to humans and has been used for research and training in other studies [26-30].

## Conclusions

This pilot study shows that the pancreas ligation band may prevent POPF after distal pancreatectomy by atraumatic ligation and stenosis of the pancreatic duct and that the band ligates the pancreas while maintaining arterial blood flow and reducing pancreatic necrosis. The linear stapler cannot close the pancreas without trauma and cannot adjust arterial blood flow. The pancreas ligation band will become an alternative treatment if it proves superior to the linear stapler in preventing POPF in distal pancreatectomy. Further studies are needed to compare the incidence of POPF after using the ligation band and the linear stapler.
